# A Simulation Study to Reveal the Epidemiology and Aerosol Transmission Characteristics of *Botrytis cinerea* in Grape Greenhouses

**DOI:** 10.3390/pathogens13060505

**Published:** 2024-06-13

**Authors:** Lifang Yuan, Hang Jiang, Tinggang Li, Qibao Liu, Xilong Jiang, Xing Han, Yanfeng Wei, Xiangtian Yin, Suna Wang

**Affiliations:** 1 Shandong Academy of Grape, Shandong Academy of Agricultural Sciences, Jinan 250100, China; yuanlifang@saas.ac.cn (L.Y.); liuqibao@saas.ac.cn (Q.L.); hanxing@nwafu.edu.cn (X.H.); weiyanfeng@saas.ac.cn (Y.W.); 2 Institute of Plant Protection, Shandong Academy of Agricultural Sciences, Jinan 250100, China; 3 School of Landscape and Ecological Engineering, Hebei University of Engineering, Handan 056038, China

**Keywords:** airborne transmission, aerosol, grape gray mold, *Botrytis cinerea*, quantitative detection

## Abstract

Most previously studies had considered that plant fungal disease spread widely and quickly by airborne fungi spore. However, little is known about the release dynamics, aerodynamic diameter, and pathogenicity threshold of fungi spore in air of the greenhouse environment. Grape gray mold is caused by *Botrytis cinerea*; the disease spreads in greenhouses by spores in the air and the spore attaches to the leaf and infects plant through the orifice. In this study, 120 μmol/L propidium monoazide (PMA) were suitable for treatment and quantitation viable spore by quantitative real-time PCR, with a limit detection of 8 spores/mL in spore suspension. In total, 93 strains of *B. cinerea* with high pathogenicity were isolated and identified from the air samples of grapevines greenhouses by a portable sampler. The particle size of *B. cinerea* aerosol ranged predominately from 0.65–3.3 μm, accounting for 71.77% of the total amount. The *B. cinerea* spore aerosols were infective to healthy grape plants, with the lowest concentration that could cause disease being 42 spores/m^3^. *Botrytis cinerea* spores collected form six greenhouse in Shandong Province were quantified by PMA-qPCR, with a higher concentration (1182.89 spores/m^3^) in May and June and a lower concentration in July and August (6.30 spores/m^3^). This study suggested that spore dispersal in aerosol is an important route for the epidemiology of plant fungal disease, and these data will contribute to the development of new strategies for the effective alleviation and control of plant diseases.

## 1. Introduction

Grapevine (*Vitis vinifera* L.) is widely cultivated worldwide, with a cultivation area of 6.95 million hectares and 78.03 million tons of production (FAOSTAT 2020; data available at http://faostat.fao.org/, accessed on 23 December 2023). In China, it was reported that the cultivation areas of fresh table grapes and wine grapes were 730.7 thousand hectares and 80 thousand hectares, respectively, in 2021. Greenhouse planting is the main mode of fresh table grape production in China, which increases the occurrence of grape gray mold because of the high humidity. Grape gray mold is caused by *B. cinerea*, and the disease can infect the inflorescence, young fruit and ripe fruit, leading to a yield loss of 20–60% [1,2,3,4]. In addition, the majority of tested grape cultivars were susceptible to *B. cinerea*, which made it a vital limiting factor for grape production [4,5].

*Botrytis cinerea* is a necrotrophic fungus that can infect more than one thousand known hosts, including dicotyledonous and monocotyledonous plants, where it infects vegetables, fruits, flowers, rotting vegetables, and postharvest fruits and flowers to produce a dense gray lawn of new conidia [6,7]. *Botrytis cinerea* can survive as mycelia, conidia, or sclerotia in crop debris for long periods, for example, 30 weeks in grape vine pruning [7]. The high adaptability of *B. cinerea* to different environments have allowed it to spread widely across the plant kingdom, although *B. cinerea* strains exhibit considerable variability in colony morphology, mycelial growth, conidiation, and sclerotium formation [6,8,9]. The conidia of *B. cinerea* are spread locally by wind and water droplets, and some insects can disseminate them efficiently [10,11,12]. Conidia are the primary inoculum of gray mold; an abundant and constant availability of viable conidia occurs in the air from the beginning of spring, and the highest conidial concentrations are observed in warm and humid weather [10,13]. Many genetic, physical, and environmental factors, including grape cultivar, morphological, and physiological features of the berry skin of the grape, wound features, temperature. and humidity, are key to the promotion of grape gray mold outbreaks [4,13,14,15,16]. Considering the size of *B. cinerea* spores (10–12 × 8–10 μm), spore infection of the stoma in grape (23–28 × 15–18 μm) occurs easily [13,17].

Bioaerosols are aerosols that are composed of bacteria, fungal spores and hyphae, viruses, cellular fragments, and pollen [18,19]. Bioaerosols are important vectors for the transmission of various infectious diseases of humans, nonhuman animals, and plants and can cause disease outbreaks [20]. Bioaerosols transmission is regarded as an important route in the epidemiology of plant disease; the emission and transmission of *Pseudomonas amygdali* pv. *lachrymans* aerosol is a significant cause of cucumber angular leaf spot in greenhouses [21]. Airflow, soil, water, seeds, and rainfall are major modes for plant fungal disease dispersal [22,23,24,25]. Airborne fungi are widely distributed in the environment, and it is commonly believed that airborne fungi pose serious health risks in crops, such as rice blast caused by *Magnaporthe oryzae* and wheat stem rust caused by *Puccinia graminis* f. sp. *tritici* [26,27]. Airborne diseases differ widely in transmissibility, and they are most easily transmitted over a short range based on the higher concentration of pathogen-containing aerosols closer to infected plants [21,28]. The airborne conidia of grape gray mold caused by *B. cinerea*, as a disease with airborne transmission, were detected and quantified as 495 spores/m^3^ in the grapevine flowering period in Cenlle in northwest Spain in 2018 [29,30]. However, a major problem is the lack of threshold values for *B. cinerea* in greenhouse vineyards predicting outbreaks of the disease, which is valuable for epidemiological forecasting and control of the diseases.

Considering the airborne gray mold to infect various crops worldwide, it is urgent and important to understand the extent of the risk associated with the airborne transmission of the disease in greenhouses. The aims of this study were as follows: (i) to establish a highly effective method for collecting and quantitatively detecting the live spores of *B. cinerea* in bioaerosol; (ii) to determine the dynamics and size distribution of *B. cinerea* aerosols released by artificially infested grape plants; and (iii) to determine the threshold values of *B. cinerea* conidia in greenhouse vineyards for grape gray mold outbreaks. These data will provide insight into the occurrence and epidemiology of grape gray mold in greenhouses, which will support the development of effective strategies for forecasting and limiting the spread of the airborne disease in greenhouses elsewhere.

## 2. Materials and Methods

### 2.1. Air Sample Collection, Fungal Isolates, and Culture Conditions

A portable sampler (HighBioTrap, Beijing Blue Tech, Inc., Beijing, China) was used to collect air samples at 1.5 m above the ground in grapevine greenhouses [19,21]. The grapevine greenhouses in China were located in Jiyang district in Jinan city, Dahu town in Zhaoyuan city. Aerosol samples were collected during April to September. For each sampled greenhouse, air samples were collected every hour during 9:00~17:00. Three adjacent greenhouses were used for samples collection (Table 1). For each sampling time point, air samples were collected onto 90 mm (diameter) sterilized aluminum membranes coated with 600 mL of mineral oil and PDA (200 g of potato, 20 g of glucose, and 20 g of agar per liter) medium for 1 min. After sampling, all PDA plates were cultured at 20 °C for 48 h and total fungal strains were isolated and purified for culture and further identification. Air samples collected on the aluminum membranes were assessed by propidium monoazide (PMA)-qPCR.

### 2.2. Identification of B. cinerea and Pathogenicity

The strains incubated on PDA at 20 °C were used for morphological identification. The morphological features and the size of the conidia (length and width, *n* = 50) were observed and measured photographed with a microscope (Olympus, Hamburg, Germany). To further confirm the fungal species, the internal transcribed spacer (ITS) region of the ribosomal DNA was amplified using the primers ITS1/ITS4, and the sequences were compared to type sequences in GenBank. In the pathogenicity tests, detached leaves of grape (cv. Red globe, 1 year old) were inoculated with mycelium plugs (5 mm diameter) (from 3-day-old isolate cultures), and controls were inoculated with PDA plugs (5 mm diameter). All treated grape leaves were placed in a greenhouse maintained at 25 °C and 95% relative humidity. The infection rate and lesion diameter were measured 3 days after inoculation.

### 2.3. Establishment of PMA-qPCR for the Detection of Live Spores of B. cinerea

The primer pair P5-F (5′-GGAAGGATCATTACAGAGTTCATG-3′) and P5-R (5′-GGTATTCTCTGGCGAGCATACAAGG-3′) was designed for specific amplification of *B. cinerea* DNA based on sequence alignments of the ITS gene from *B. cinerea*, common pathogens (*Botryosphaeria dothiea*, *Alternaria tenuissima*, *Colletotrichum gloeosporioides*, *Sphaceloma ampelium*, *Uncinula necator*, *Fusarium graminearum*, *Agrobacterium tumefaciens*, *Allorhizobium vitis*) on grape, and nonpathogenic fungus (*Alternaria solani*, *A. tenuissima*, *A.alternata*, *Penicillium oxalicum*, *A. seleniiphila*, *Aspergillus niger*, *A.alternata*, *Neurospora* sp., *Fusarium equiseti*) isolated from the air in vineyards, as available in the NCBI database (Figure S1). The specificity of the primers P5-F and P5-R was studied by conventional PCR and real-time PCR by using *B. cinerea*, common pathogens on grape and nonpathogenic fungus isolated from the air in vineyards.

Real-time PCR reactions was performed in a 20 μL volume, including 1 μL template DNA (10 ng), 10 μL of 2 × AceQ Universal SYBR qPCR master Mix (Vazyme), and 0.4 μL of each 10 μmol/L primer (P5-F, P5-R). qRT-PCR was performed in a Roche LightCycler 480; the conditions were 95 °C for 5 min, followed by 40 cycles of 95 °C for 10 s and 60 °C for 30 s. A melting curve analysis was performed from 62 °C to 95 °C, with a 0.5 °C/30 s increment. Cycle threshold (Ct) values were calculated automatically by Roche LightCycler 480 software. A standard curve for the quantification of *B. cinerea* was generated by analyzing a 10-fold dilution series of *B. cinerea* spore suspensions from 8 × 10^7^ to 8 spores/mL [31].

To optimize the PMA (Biotium, Inc., Hayward, CA, USA) treatment for quantifying viable *B. cinerea* spores, PMA was added to viable or dead *B. cinerea* spores derived from suspensions of 8 × 10^7^ to 8 spores/mL with final concentrations of 0, 20, 40, 60, 80, 120, and 140 μmol/L. Then dark treatment for 15 min to allow the PMA to penetrate into dead spores, and 50 W LED light treatment for 10 min as Meng distributed [32]. After centrifugation (12,000× *g*, 1 min), 1 μL of supernatant containing the DNA was used directly in qRT-PCR.

### 2.4. Quantitative Detection of B. cinerea in Naturally Infested Grapevines

Air samples were collected from greenhouses located in Jiyang district, Dahu town, and aluminum membranes for collecting air samples were used to analyze the live spores of *B. cinerea*. The mineral oil membrane was washed with Tween 20 suspension and then transferred to a 50 mL centrifuge [21]. To remove the mineral oil, the obtained oil-in-water emulsion was centrifuged at 8000 rpm, and the pellet was resuspended in 1 mL of sterile water with PMA treatment (final concentration of 120 μmol/L), then centrifuged, after which DNA was extracted with the OMEGA fungal DNA Kit according to the manufacturer’s guidelines. Finally, real-time PCR was used to quantify the spores in infested grapevines.

### 2.5. Size Distribution and Infection Threshold of Aerosolized B. cinerea

An aerosol chamber was used to produce *B. cinerea* aerosol, which was made of organic glass with a size of 70 cm × 60 cm × 60 cm (length × width × height) [21]. Nine grape plants (cv. Red globe) were inoculated with a 1 × 10^8^ spores/mL suspension of *B. cinerea* by spraying. Grape plants treated with sterile water used as the control. Then, the plants were placed and incubated in the aerosol chamber under 90 ± 5% humidity and 25 ± 2 °C. A six-stage Andersen sampler (Thermo-Andersen, Smyrna, GA, USA) was used to collect the aerosol samples onto 90 mm diameter PDA plates at 1 to 7 days post inoculation (dpi). The colony-forming units (CFU) of *B. cinerea* were counted after culture 48 h at 20 °C. According to the inertia-related aerodynamic diameter of Andersen sampler, the size distribution of *B. cinerea* aerosols were assessed by Andersen sampler [32].

Ten-fold serial dilution of *B. cinerea* suspensions from 2.5 × 10^6^ to 2.5 × 10^1^ spore/mL were aerosolized into the aerosol chamber by a six-jet Collison nebulizer (BGI Inc., Waltham, USA) at a flow rate of 12 L/min (pressure of 20 psi) for 2 h [33]. The aerosol samples were collected onto 90 mm diameter PDA plates by an Andersen sampler for measure the number of the colony-forming units (CFU) of *B. cinerea*. The initial concentration of *B. cinerea* aerosol was determined 30 min after nebulization. The aerosol samples were collected onto 90 mm diameter PDA plates by an Andersen sampler and cultured at 20 °C for 48 h; then, the PDA colonies were enumerated. Grape seedlings were kept at 90 ± 5% RH and 25 ± 2 °C, and disease development was observed every day. The incidence rate (IR) was examined at 7 dpi.

### 2.6. Statistical Analysis

Statistical analyses were completed with SPSS 22.0 software, all experiments were repeated at least three times, the concentration levels of *B. cinerea* spores were recorded as the mean ± standard deviation (SD), and significance was accepted at *p* < 0.05 [34,35].

## 3. Results

### 3.1. Identification and Pathogenicity of B. cinerea Strains Isolated from the Bioaerosols in Greenhouses

**Figure 1:**
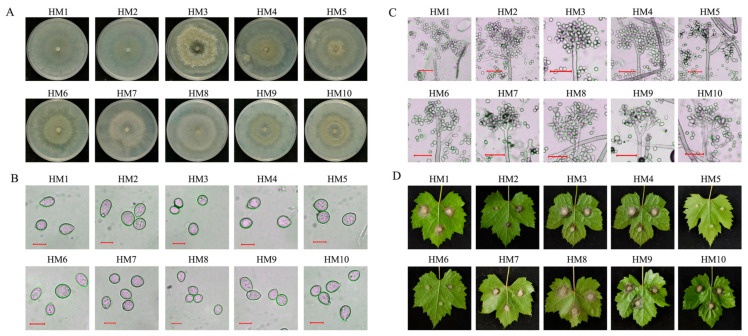
Characterization of *B. cinerea* collected in grape greenhouses. (**A**) Plate morphology of *B. cinerea* isolated from aerosol samples. *B. cinerea* on PDA plates were cultured in the dark for 7 days at 20 °C. (**B**) Conidia of *B. cinerea* strains. The bar is 10 μm. (**C**) Conidiophores of *B. cinerea* strains. The bar represents 50 μm. Plates were inoculated with hyphae of *B. cinerea* and cultured in the dark for 14 days for conidium production. Conidiophores and conidia were observed by stereomicroscopy and bright-field light microscopy. (**D**) The pathogenicity of *B. cinerea* strains.

The bioaerosols were sampled by a portable sampler from 26 April to 26 September in 2022 in Jiyang district and Dahu town. In total, 93 of the 756 strains isolated from bioaerosol samples were identified as *B. cinerea* on the basis of morpho-cultural characteristics and molecular analysis of the ITS sequences, and the ITS sequences were 99.80%, 99.80%, and 99.60% identical to the sequence data from *B. cinerea* isolates in GenBank (BC, SJH-2 and ASF-Bc1, respectively) (Table 1 and Figure S3). The conidia were ellipsoid or ovoid and measured 8.5–15.6 × 8.1–12.8 μm (*n* = 50). The conidiophores were straight, septate, branched, and pale brown to brown and measured 450–1350 × 13–20.6 μm (*n* = 20) (Figure 1B,C). Pathogenicity tests were conducted on detached leaves of grape, and all of the strains were found to infect grape leaves, although with slightly different degrees of virulence (Table 1).

### 3.2. Establishment of PMA-qPCR for the Detection of Live Spores of B. cinerea in the Air

#### 3.2.1. Specificity of the Primers

**Figure 2:**
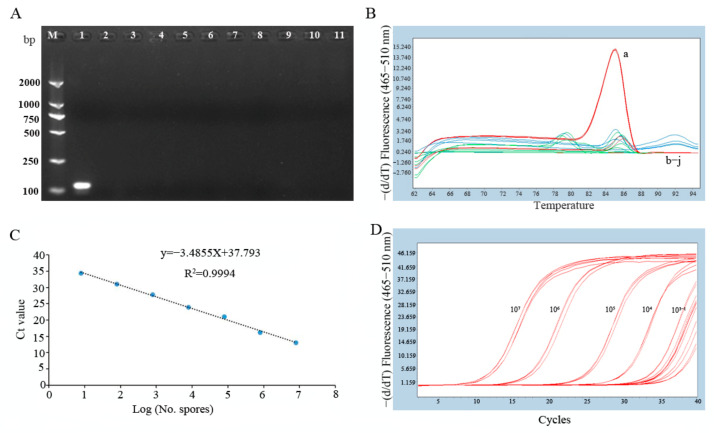
Specificity of the primers and amplification. (**A**) Specificity detection of *B. cinerea* with the primer P5. Lane M. marker; Lane 1. *B. cinerea*; Lane 2. *Coniella vitis*; Lane 3. *Botryosphaeria dothiea*; Lane 4. *Alternaria tenuissima*; Lane 5. *Colletotrichum gloeosporioides*; Lane 6. *Sphaceloma ampelium*; Lane 7. *Uncinula necator*; Lane 8. *Fusarium graminearum*; Lane 9. *Agrobacterium tumefaciens*; Lane 10. *Allorhizobium vitis*; Lane 11. negative control. (**B**) The melting curve of real-time PCR for *B. cinerea* and other species. The melting curve of *B. cinerea* has a single peak with a Tm of 85.2 °C, and those of 9 other strains have no peak or slight peaks with different Tm values. (a) *B. cinerea*. (b–j) other species of pathogenic fungi and bacteria on grape. (**C**) The standard curve and amplification curve (**D**) of real-time PCR for gradient dilution of *B. cinerea* spores. Spores were serially diluted prior to DNA extraction, and the DNA from three replicates was pooled for each point on the standard curve. Ct, cycle threshold. Points are representative of DNA extracted from (1) 8 × 10^7^, (2) 8 × 10^6^, (3) 8 × 10^5^, (4) 8 × 10^4^, (5) 8 × 10^3^, (6) 8 × 10^2^, and (7) 8 spores. Efficiency = 93.60%.

The specificity of the primers P5-F and P5-R was studied by conventional PCR and real-time PCR by using *B. cinerea*, common pathogens on grape and nonpathogenic fungus isolated from the air in vineyards. A single fragment of 115 bp was amplified from *B. cinerea* genomic DNA, and no product was amplified from isolates of 7 species of common pathogenic fungi on grape, 2 bacteria (*A. tumefaciens*, *A. vitis*), and 9 species of nonpathogenic fungi isolated from the air in vineyards (Figure 2A and Figure S2). The melting curve of *B. cinerea* had a single peak with Tm 85.2, while the melting curves of the negative control and other fungi had no peak or a slight peak with different Tm values (Figure 2B).

To study the sensitivity and standard curve of real-time PCR, a dilution series of *B. cinerea* spore suspensions from 8 × 10^7^ to 8 spores/mL was prepared. The standard curve was generated by plotting the log spores (No. spores) of *B. cinerea* against the Ct value determined by qPCR. Linearity was observed across the whole range used, and a very large correlation coefficient (R^2^ = 0.99) indicated very low intra-assay variability; the amplification efficiency was 93.60% (Figure 2D). The limit of detection was defined as the smallest population of microorganisms that could be detected by using the SYBR Green qPCP method. The detection limit of *B. cinerea* genomic DNA of the real-time PCR assay in this study was 8 spores/mL (Figure 2C), which is significantly higher than that of conventional PCR (Figue S4).

#### 3.2.2. PMA Concentration Optimization and Validation

**Figure 3:**
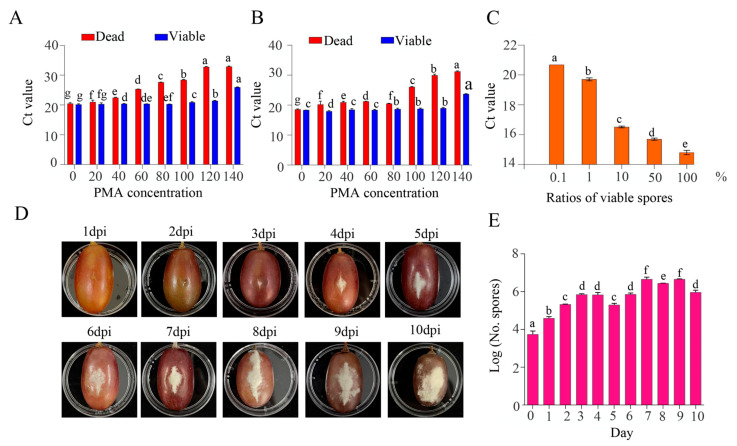
(**A**) Ct values of viable or dead spores of *B. cinerea* strains treated with different concentrations of PMA. (**A**,**B**) The results for 10^7^ spores/mL and 10^8^ spores/mL, respectively. (**C**) Detection of the defined ratio of viable spores of *B. cinerea* by PMA-qPCR methods. (**D**) The symptoms of gray mold on grape fruit after inoculation with *B. cinerea*. (**E**) Detection of viable *B. cinerea* on grape fruit infected by *B. cinerea*. Bars indicate standard error, and Different letters indicate significant differences (*p* < 0.05).

Propidium monoazide was used to bind the DNA of dead cells to ensure the DNA amplification of live cells. In this study, the results indicated that more than 120 μmol L^−1^ PMA (e.g., 140 μmol/L) affects the viability of intact spores of *B. cinerea*, and PMA can function as a disinfectant to inactivate spores of *B. cinerea*. Thus, the PMA concentration of 120 μmol/L was selected for the treatment of *B. cinerea*, which had the weakest influence on the viability of *B. cinerea* spores. Meanwhile, this concentration was most efficient for distinguishing the viable and inactivated spores of *B. cinerea* (Figure 3A,B). Furthermore, we evaluated the ability of PMA to inhibit the amplification of DNA from nonviable spores of *B. cinerea* by RT–qPCR assays of a series of mixtures of viable and inactivated spore suspensions (Figure 3C). The results showed that the Ct value increased with a decrease in the proportion of viable spores of *B. cinerea*. When the percentage of viable spores dropped from 100% to 0.1%, the Ct values of real-time PCR ranged from 14.81 to 20.70 (Figure 3C). Interestingly, the disease severity of grape increased from 1 dpi to 10 dpi, and the live spores of *B. cinerea* in fruit increased first and then decreased. The largest number of spores of *B. cinerea* in fruit (4.47 × 10^6^ spores) was detected at 9 dpi, and the number deceased at 10 dpi (Figure 3D,E).

### 3.3. Quantitative Detection of B. cinerea in the Air around Naturally Infested Grapevines

**Figure 4:**
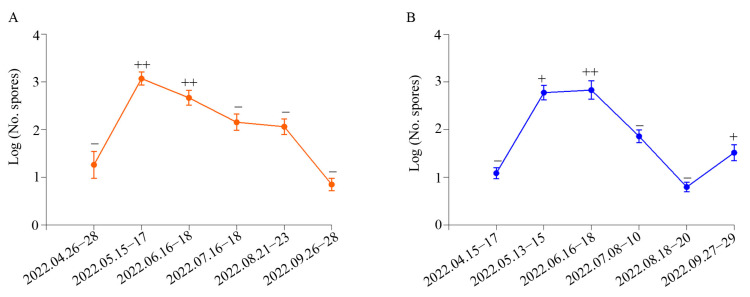
The population dynamics of *B. cinerea* in greenhouses in different periods and disease occurrence. (**A**) Jiyang district in Jinan city. (**B**) Dahu town in Zhaoyuan city. −, + and ++: Severity of Botrytis cinereal.

To examine the epidemiological dynamics and optimal preventive intervention time for grape gray mold in greenhouses, we detected *B. cinerea* spores in the air and investigated disease index of gray mold during the growing season of grapes (Figure 4A,B, Table 1). There was almost no grape gray mold in April in the greenhouse, and the spore concentration of *B. cinerea* was 12–18 spores/m^3^. The spore concentration of *B. cinerea* in the air environment was highest (466–1182 spores/m^3^) when collected in May and June during the growing season in grape greenhouses, which also demonstrated that the concentration of *B. cinerea* spores in the air leading to serious grape gray mold occurred and spread. Next, the concentration of *B. cinerea* decreased to 6 spores when detected in July and August, and the severity of grape gray mold become weak and the symptoms even disappeared. However, disease recurrence occurred in September in Dahu Village of Shandong Province, with a *B. cinerea* spore concentration of 33 spores/m^3^, but a similar result was not observed in Jiyang city of Shandong Province (Figure 4B).

### 3.4. Population Dynamics and Size Distribution of B. cinerea Aerosols Released by Infested Grape Plants

**Figure 5:**
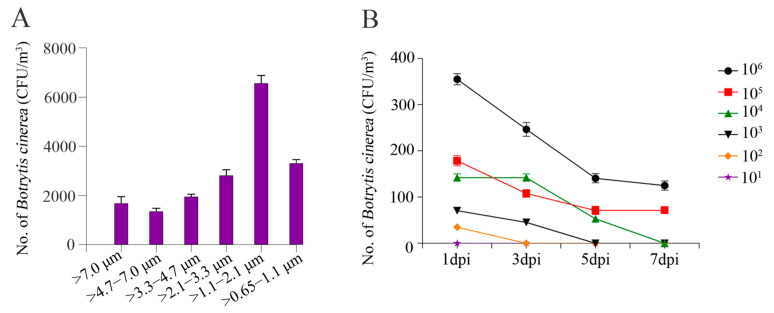
(**A**) The size distribution of *B. cinerea* aerosols and (**B**) population dynamics of *B. cinerea* aerosols released by artificially infested grape plants in the aerosol chamber. Grape plants were inoculated with *B. cinerea* by spraying different concentrations of spore suspensions. Aerosol samples were collected daily from 1 to 7 days post inoculation (dpi). Particle size was determined by a six-stage Andersen sampler. Error bars for the number of spores in aerosols represent the standard deviations (SDs) of three biological replicates.

To evaluate the size distribution of *B. cinerea* aerosols in the air in a grape greenhouse with gray mold, a six-stage Andersen sampler was used. The results showed that most of the *B. cinerea* aerosols (approximately 82.46%) were distributed between stage 4 and stage 6 (0.65–3.3 μm), while a fairly lower proportion of *B. cinerea* aerosols were detected as stage 1 (>7.0 μm) and stage 2 (4.7–7.0 μm), accounting for approximately 9.54% and 8.00%, respectively (Figure 5A).

Grape plants (one year old) inoculated with different concentrations of *B. cinerea* spore suspensions were incubated in the aerosol chamber. We detected the concentration of *B. cinerea* aerosols released from infested grape plants by artificial inoculation. The highest concentration of *B. cinerea* was detected at 1 dpi, regardless of the spore concentration of *B. cinerea* used for inoculation, and the higher the spore concentration for inoculation was, the higher the amount of *B. cinerea* released in the aerosol chamber. The concentration of *B. cinerea* in the air decreased significantly beginning at 5 dpi (Figure 5B). In addition, no *B. cinerea* was detected in the aerosol chamber for grape plants inoculated with 10 spores/mL (Figure 5B).

### 3.5. The Pathogenicity of Aerosolized B. cinerea and the Infection Threshold

**Figure 6:**
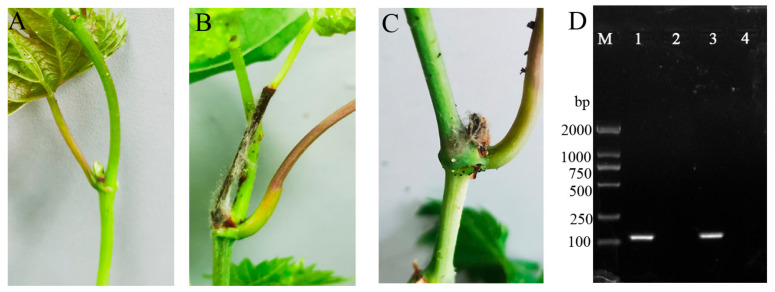
(**A**) Healthy grape plants. (**B**) Symptoms observed on *B. cinerea*-infected grape plants inoculated with 10^8^ spores/mL suspension at 7 dpi. (**C**) Symptoms on grape plants inoculated with *B. cinerea* aerosols at 7 dpi. (**D**) Detection of *B. cinerea* in diseased leaves of grape plants by PCR with the P5 primers. Lane M, marker; Lane 1, genomic DNA from leaves of *B. cinerea*-infected grape plants; Lane 2, genomic DNA from leaves of control healthy plants; Lane 3, genomic DNA from leaves of grape plants inoculated with *B. cinerea* aerosol; Lane 4, negative control.

To determine the pathogenicity of aerosolized *B. cinerea*, we used a six-jet Collison nebulizer to produce *B. cinerea* aerosols. The Collison nebulizer was connected to the air inlet of the aerosol chamber. *B. cinerea* spores were prepared and aerosolized into the aerosol chamber at a flow rate of 12 L/min (pressure of 20 psi) for 2 h. Grape plants were exposed to aerosolized *B. cinerea* spores, and the corresponding incidence rate (IR) values were determined. The results showed that aerosolized *B. cinerea* could cause serious grape gray mold (IR > 30%) when the grape plants were exposed to a higher concentration of aerosolized *B. cinerea* spores (>42 spores/m^3^), which was produced with spore suspensions of 10^6^, 10^5^, and 10^4^ spores/mL (Table 2). The infected shoots of grape plants turned brown, and the tissues became soft and exhibited a gray mold layer (Figure 6A–D). The IR decreased with a lower concentration of aerosolized *B. cinerea* in the aerosol chamber when the concentration of aerosolized *B. cinerea* spores was lower than 16.49 spores/m^3^, and no visible symptoms were observed on grape plants when the inoculation concentration was lower than 2.5×10^3^ spores/mL.

## 4. Discussion

Fungal aerosols are ubiquitous in the atmosphere and play an important role in global climate change and human and nonhuman animal health (e.g., *Aspergillus fumigatus*), and the size distribution and concentration of fungal aerosols are key for infection of the host [36,37,38,39,40,41]. Airborne transmission has also been considered an important mode for the epidemiology of plant fungal disease, in addition to water, insects, and animals, for dispersal of their spores, e.g., in *Corynespora cassiicola*, *Alternaria alternate*, and *P. graminis* f.sp. *tritici* [26,28,42]. Gray mold is an important fungal disease responsible for grape losses in most vineyards worldwide, and *B. cinerea* has been reported as an airborne fungus that spreads disease [29]. However, most studies have provided insights into the role of fungal aerosols in the transmission of plant diseases based on traditional spore trapping and morphological observations [29,43]. Recently, the detection of bioaerosols has facilitated plant disease transmission studies with the development of new air sampling and molecular detection technologies. In this study, an Andersen sampler and a portable sampler were used for size distribution analysis, and *B. cinerea* was quantified by PMA-qPCR.

In our previous study, *B. cinerea* spores were detected in a grape greenhouse with gray mold, and *B. cinerea* strains isolated from air samples collected in greenhouses were infective toward grape plants. These results demonstrate that *B. cinerea* in bioaerosols could spread grape gray mold, although wind, rain, insects, and farm implements could be the method for transport of the fungi [44,45]. In this study, *B. cinerea* aerosols were produced by a six-jet Collison nebulizer in exposure chambers to avoid other transmission routes, and the results proved that *B. cinerea* aerosols instead of other transmission factors cause grape gray mold. Fungi in bioaerosols are generally derived from soil, water, plants, animals, and so on [46,47]. Temperature, humidity, wind, and sea ice are important meteorological conditions that influence the concentrations and sizes of airborne fungi [46,48]. Meanwhile, temperature and humidity are the major factors affected the survival, metabolism, production, and release of fungi spores [49,50,51]. For example, sporulation of *Coniella diplodiella* occurred between 10 °C and 35 °C, with the optimum detected at 20 °C [50]. In addition, wind speeds also affected the dispersal of fungi spores in aerosols [52]. In our study, we found that the concentration of *B. cinerea* in the air in grape greenhouses was higher in May and June and lower in July and August; meanwhile, grape gray mold was barely observed in the field in July and August. The results suggested that the higher temperature may affect the activity of *B. cinerea* in aerosols, which leads to a lower transmission risk and lower disease severity.

Aerodynamic diameter is a commonly used size measure for airborne particles [46]. The size of bioaerosols is a main factor that impacts their ability to reach plant tissues through natural openings and successfully infect host plants [21]. Previous studies showed that the size distributions of airborne fungi ranged from 0.65 µm to larger than 7 µm [39,47]. In our study, approximately 38% of *B. cinerea* aerosols were distributed in the 1.1–2.1 μm range, and particle sizes of 4.7–7.0 µm were the least frequent (accounting for 7.6%). Previous studies have shown that the stoma of grape leaves is 15.2–35.2 μm [53]. In addition, the waxy layer on the surface of berries is thin, and the lamella structure of berries can be destroyed into an amorphous form when berries are in contact with each other, which results in holes with a diameter of 0.5–2.0 mm. These data indicated the possibility for aerosol transmission of grape gray mold.

The plant canopy is the main source of bioaerosols, and most pathogenic bacteria and fungal aerosols (e.g., *P. amygdali* pv. *lachrymans* aerosol, *P. syringae* pv. *glycinea* aerosol, *C. cassiicola*) are commonly released from host plants [21,28]. However, the dynamics of *B. cinerea* in the air released from infected grape and the threshold concentration of *B. cinerea* aerosol causing grape gray mold are largely unknown. In this study, the release peak of *B. cinerea* aerosol was detected one dpi, and the release decreased with time. Additionally, the release concentration increased with the inoculation concentration of *B. cinerea* spores, and *B. cinerea* in the air at 1 dpi was detected at a concentration of 357 CFU/m^3^ with an inoculation of 10^6^ spores/mL. The aerosol inoculation test in the aerosol chamber showed that the lowest concentration of *B. cinerea* aerosol that could cause gray mold on grape plants was 45.57 CFU/m^3^.

## 5. Conclusions

Our study demonstrated that grape gray mold in greenhouses can be spread by *B. cinerea* aerosol, and PMA-qPCR assay was developed for quantifying viable *B. cinerea* spores in the air of the greenhouse. The canopies of diseased grape plants served as the sources of *B. cinerea* aerosol, with a higher concentration (1182.89 spores/m^3^) in May and June and a lower concentration in July and August (6.30 spores/m^3^). The particle size of *B. cinerea* aerosol was mainly distributed between 0.65 to 3.3 μm, and the infection threshold of *B. cinerea* aerosol was 45.57 CFU/m^3^. Regular *B. cinerea* aerosol monitoring in grape greenhouses should be established considering the early warning and control of grape gray mold.

## Figures and Tables

**Table 1:** Sampling and identification of *B. cinerea* in bioaerosol of a naturally infested greenhouse.

Jianan City						Zhaoyuan City					
Sample No.	Greenhouse No.	Sampling Time	Disease Index (DI)	No. *B. cinerea* Isolated	Pathogenicity	Sample No.	Greenhouse No.	Sampling Time	Disease Index (DI)	No. *B. cinerea* Isolated	Pathogenicity
1	I	2022.4.26	3.27	3	+	19	IV	2022.4.15	3.98	2	+
2	II	2022.4.27	2.35	2	+	20	V	2022.4.16	4.02	1	+
3	III	2022.4.28	4.33	2	+	21	VI	2022.4.17	2.19	0	/
4	I	2022.5.15	14.40	2	+	22	IV	2022.5.13	18.89	3	+
5	II	2022.5.16	19.86	3	+	23	V	2022.5.14	16.01	5	+
6	III	2022.5.17	18.98	7	+	24	VI	2022.5.15	15.23	1	+
7	I	2022.6.16	17.34	2	+	25	IV	2022.6.16	18.65	2	+
8	II	2022.6.17	21.98	7	+	26	V	2022.6.17	15.98	3	+
9	III	2022.6.18	18.45	3	+	27	VI	2022.6.18	18.56	2	+
10	I	2022.7.16	6.16	6	+	28	IV	2022.7.08	10.79	1	+
11	II	2022.7.17	9.08	4	+	29	V	2022.7.09	7.67	1	+
12	III	2022.7.18	7.23	5	+	30	VI	2022.7.10	8.95	0	/
13	I	2022.8.21	9.17	4	+	31	IV	2022.8.18	8.93	1	+
14	II	2022.8.22	8.32	1	+	32	V	2022.8.19	10.22	2	+
15	III	2022.8.23	7.76	3	+	33	VI	2022.8.20	9.13	1	+
16	I	2022.9.26	7.77	4	+	34	IV	2022.9.27	13.13	4	+
17	II	2022.9.27	6.04	0	/	35	V	2022.9.28	14.19	3	+
18	III	2022.9.28	3.65	2	+	36	VI	2022.9.29	9.67	1	+

Pathogenicity results are scored as ‘+’ for infection and disease symptoms and ‘/’ for no *B. cinerea* strains used for pathogenicity test.

**Table 2:** The threshold for aerosolized *B. cinerea* infection of grape.

Concentration of *B. cinerea* Suspension (spores/mL)	Concentration of *B. cinerea* Aerosol (CFU/m^3^)	Incidence Rate (%)
2.5 × 10^6^	219.08 ± 3.53 ^a^	60.83 ± 1.44
2.5 × 10^5^	77.74 ± 7.07 ^b^	43.47 ± 1.68
2.5 × 10^4^	42.40 ± 6.42 ^b^	29.48 ± 1.30
2.5 × 10^3^	16.49 ± 2.04 ^c^	0
2.5 × 10^2^	2.36 ± 1.99 ^d^	0
2.5 × 10^1^	1.18 ± 1.78 ^d^	0
CK	/	0

Different letters indicate significant differences (*p* < 0.05).

## Data Availability

Data are contained within the article and Supplementary Materials.

## Supplementary Materials

The following supporting information can be downloaded at: https://www.mdpi.com/article/10.3390/pathogens13060505/s1, Figure S1: Sequence alignments of the ITS gene from *B. cinerea*, 7 species of common pathogenic fungi on grape (A) and 8 species of nonpathogenic fungi isolated from the air in vineyards (B). Figure S2: (A) Specificity detection of *B. cinerea* with the nonpathogenic fungus isolated from the air in vineyards using the primer P5. Lane M. marker; Lane 1. *B. cinerea*; Lane 2. *Alternaria solani*; Lane 3. *A. tenuissima*; Lane 4. *A.alternata*; Lane 5. *Penicillium oxalicum*; Lane 6. *A. seleniiphila*; Lane 7. *Aspergillus niger*; Lane 8. *A.alternata*; Lane 9. *Neurospora* sp.; Lane 10. *Fusarium equiseti*. (B) Amplification results of 10 different strains of *B. cinerea*. Lane M. marker; Lane 1–10. Represent 10 different strains of *B. cinerea.* Figure S3: Phylogenetic tree for the *B. cinerea* strains. The phylogenetic tree was constructed based on ITS gene sequences using the maximum likelihood method in MEGA 6.0. Bootstrap values (1000 replicates) are shown at the branch points. The scale bar indicates 0.05 nucleotide substitutions per nucleotide position. GenBank accession numbers associated with strains can be found at the right side of the strain number. The *B. cinerea* strains isolated from the air in grape greenhouses were labeled in red. Figure S4: The sensitivity of conventional PCR in detecting *B. cinerea*. Line M, marker; Line 1–Line 7 were representative of DNA extracted from 8 × 10^7^–8 spores. Line 8 was negative control.
