# Supplementary figures and images for: A Simulation Study to Reveal the Epidemiology and Aerosol Transmission Characteristics of Botrytis cinerea in Grape Greenhouses
Source: Pathogens. 2024 Jun 13;13(6):505. doi: 10.3390/pathogens13060505 (PMC11207035; doi:10.3390/pathogens13060505)

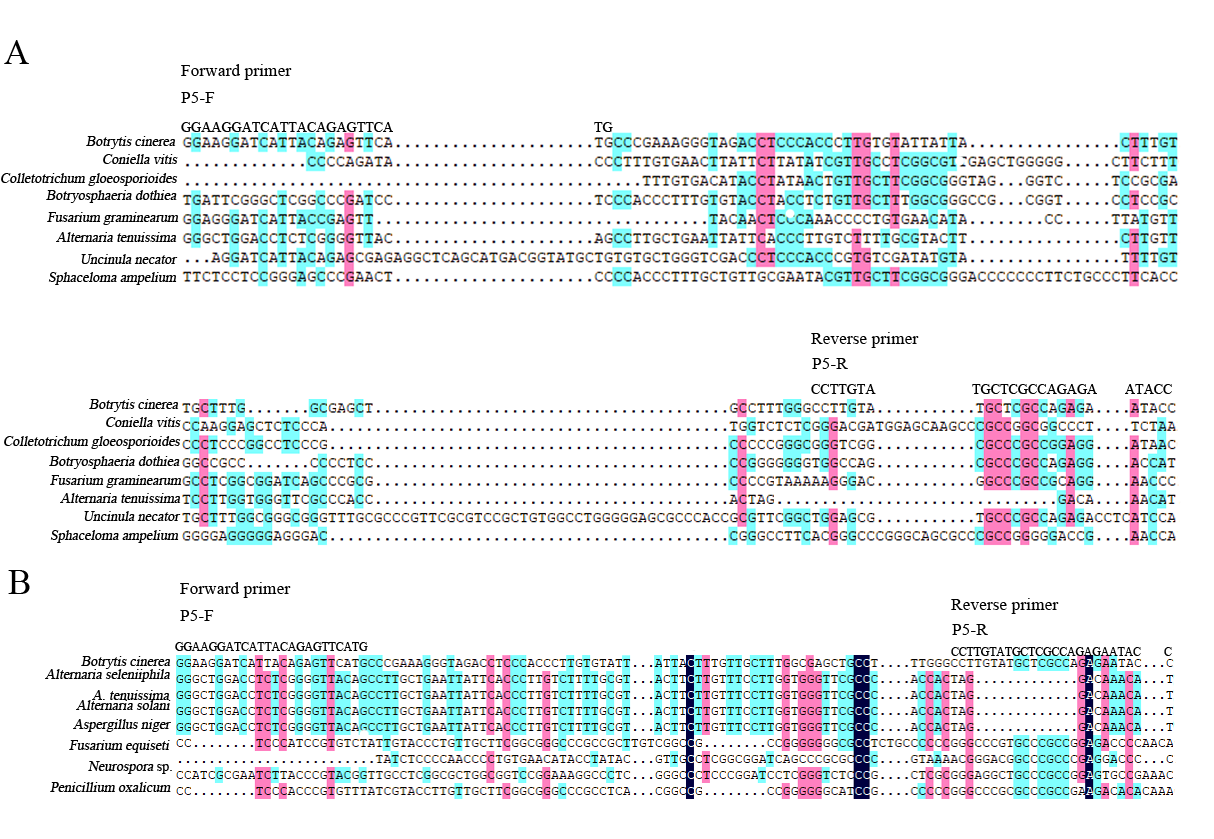

Supplement: Supplementary file 1 [file pathogens-13-00505-s001.zip › Figure S1.tif]

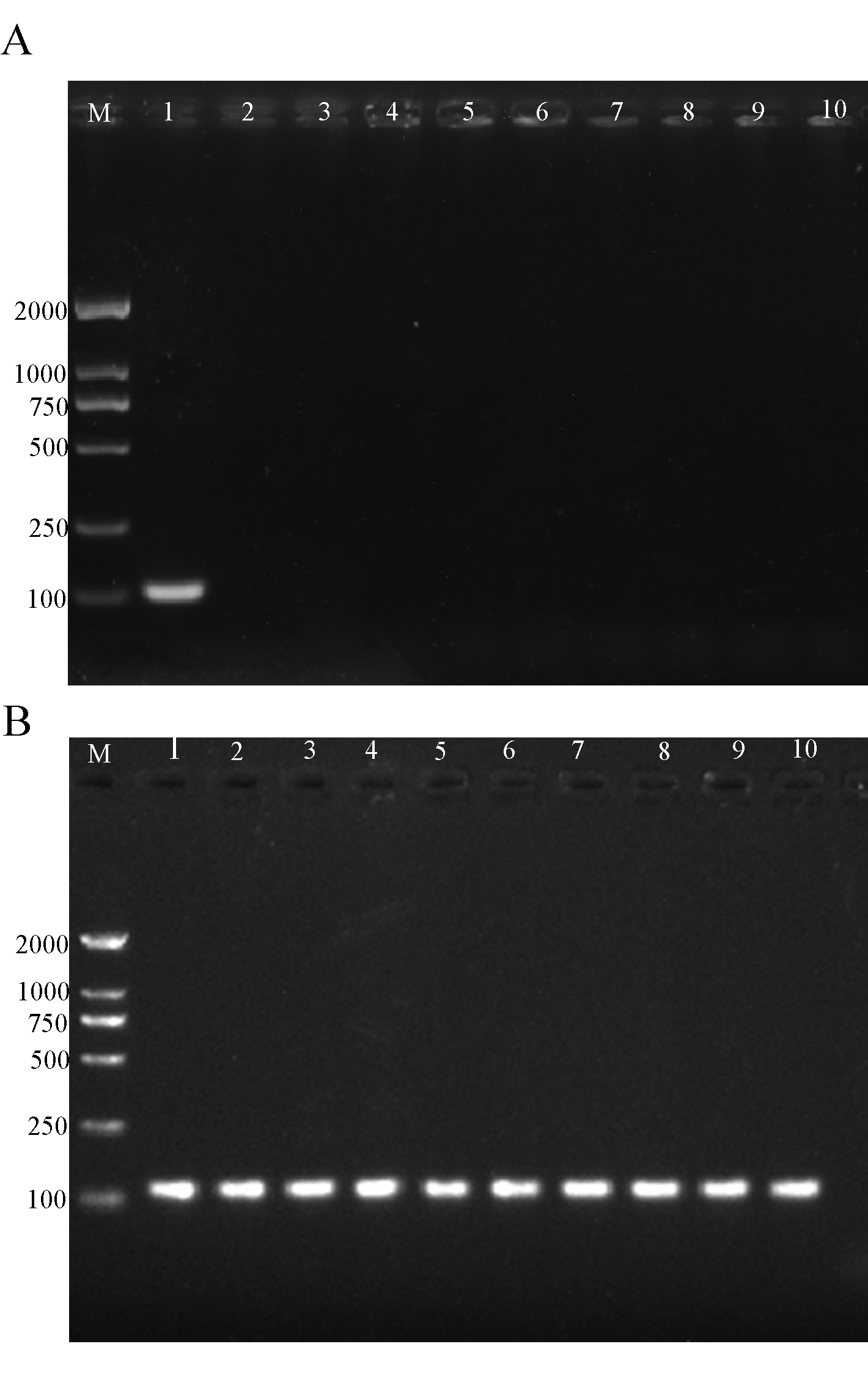

Supplement: Supplementary file 1 [file pathogens-13-00505-s001.zip › Figure S2.tif]

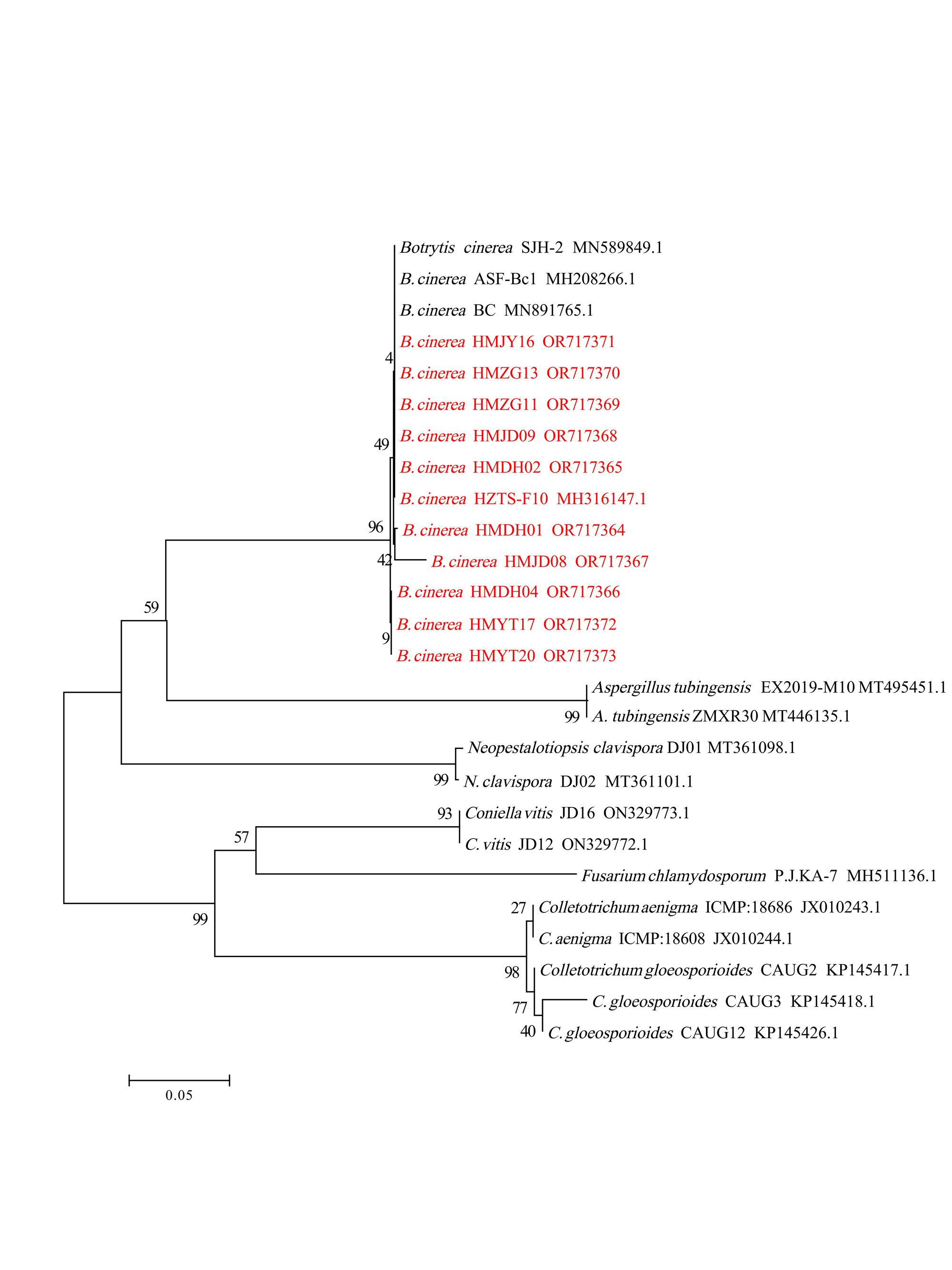

Supplement: Supplementary file 1 [file pathogens-13-00505-s001.zip › Figure S3.jpg]

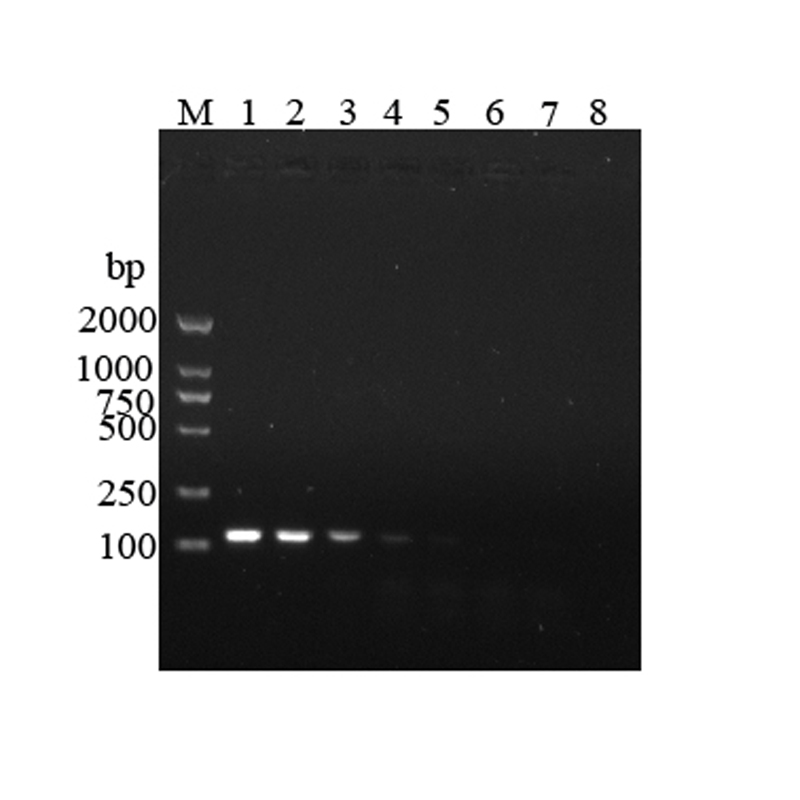

Supplement: Supplementary file 1 [file pathogens-13-00505-s001.zip › Figure S4.jpg]
